# Understanding the Morpho-Anatomical, Physiological, and Functional Response of Sweet Basil to Isosmotic Nitrate to Chloride Ratios

**DOI:** 10.3390/biology9070158

**Published:** 2020-07-08

**Authors:** Giandomenico Corrado, Luigi Formisano, Veronica De Micco, Antonio Pannico, Maria Giordano, Christophe El-Nakhel, Pasquale Chiaiese, Raffaele Sacchi, Youssef Rouphael

**Affiliations:** Department of Agricultural Sciences, University of Naples Federico II, 80055 Portici (NA), Italy; lu.formisano@gmail.com (L.F.); veronica.demicco@unina.it (V.D.M.); antonio.pannico@unina.it (A.P.); maria.giordano@unina.it (M.G.); nakhel_christophe@hotmail.com (C.E.-N.); pasquale.chiaiese@unina.it (P.C.); raffaele.sacchi@unina.it (R.S.)

**Keywords:** ionic concentration, nutrient solution, anion antagonism, growth, anatomy, physiology, aromatic profile

## Abstract

Sweet basil (*Ocimum basilicum* L.) is a leafy green with a short-production cycle that is emerging as a model species among aromatic plants. Modulating the mineral composition of the nutrient solution has proved to be a valuable tool to uncover the mechanisms and responses that higher plants adopt in relation to the availability of mineral nutrients. The aim of this work was to examine the effects on basil of four isosmotic nutrient solutions with different nitrate to chloride ratios. These two anions share uptake and transport mechanisms in plants and are often considered antagonist. To this goal, we analyzed morpho-anatomical and physiological parameters as well as quality-related traits, such as the antioxidant capacity, the leaf color, the mineral composition, and the aromatic profile in relation to the nutrient ratios. Moreover, using a full factorial design, we analyzed leaves in two consecutive harvests. The data indicated a broad, multifaceted plant response to the different nutritional ratios, with almost all the recorded parameters involved. Overall, the effects on basil can be explained by considering an interdependent combination of the nitrate and chloride roles in plant nutrition and physiology. Our work revealed the extent of the modification that can be achieved in basil through the modification of the nutrient solution. It also provided indications for more nutrient efficient growing conditions, because a moderate increase in chloride limits the expected negative impact of a sub-optimal nitrate fertilization.

## 1. Introduction

Basil (*Ocimum basilicum* L.) is an herbaceous species with a short-production cycle, appreciated as leafy green and aromatic plant [1,2]. A characterizing feature is that the secretory glandular trichomes of the basil leaves accumulate an essential oil giving the plant its easily recognizable, composite flavor [3,4]. Differently from many other spices, basil is not only used as food garnish and it is often consumed fresh. Therefore, this herb is also highly valued in different dietary regimes for its antioxidant capacity and as a source of mineral elements [1,5]. Finally, other uses of *Ocimum* spp. are in the perfume and cosmetics industry, in food processing or as folk medicine [2,6].

As any other complex trait, the nutritional and the functional quality of basil, including flavor, depends on genetic (e.g., the variety) and environmental factors, and their interaction. Especially for green vegetables, the growing conditions and the time of the leaves’ harvest are central to manage crop quality. Among pre-harvest factors, plant nutrition is arguably the most important and easily tractable element that governs growth, yield, and the phytochemical composition of a plant.

In recent years, the modulation of the mineral nutrient solutions in soilless cultivation has been increasingly applied as a tool to study the various adaptive mechanisms to nutrient availability in higher plants [7]. Moreover, these studies also gained popularity in addressing environmental issues (e.g., soil and groundwater contamination) [7]. A wealth of knowledge has been gained especially in relation to the availability of essential minerals (e.g., nutrient limitation) or toxic elements (e.g., excessive amounts). It is long established that plant uptake and use of different ions depend on their relations yet, relatively little is known on the morphological and physiological responses (e.g., indexes of growth, leaf gas exchanges, ionic accumulation, production of secondary metabolites, etc.) to the concurrent modulation of ions in nutrient solutions with identical electrical conductivities (“ionic strength”). Moreover, understanding the effect of isosmotic variations in the nutrient solution (NS) provides knowledge to alter the phytochemical profile of a plant, an area of increasing importance also for research dietitians and the food industry [8,9].

The selective uptake and mobilization of nutrients by plants is often shaped by counterions and ion antagonisms. For instance, it has been long known that nitrate and chlorine have a competitive anionic interaction that in some cases can lead to nutritional imbalance [10,11]. These two anions have similar physical and osmotic properties, and share uptake and transport mechanisms in higher plants [12,13,14]. This anion-anion interaction can be exploited to deliver valuable outcomes. For instance, nitrate uptake and accumulation in vegetables can be limited by high amount of chlorine [15,16]. On the other hand, nitrate can be exploited to mitigate chloride toxicity in Cl-sensitive crops [17,18]. Chloride is a plant micronutrient and since deficiency in soil rarely occurs, it has traditionally attracted attention as a potential toxic element when present at concentrations that exceed those required by a plant species (e.g., in saline environment). While it is well established that chloride excess limits yield [19], recent studies also underlined the beneficial functions of this microelement, present also when it accumulates in an amount typical of a macronutrient [14,19]. In particular, chloride may positively influence nitrogen use efficiency and water-holding capacity, being also an effective osmoticum [14,20]. It has been shown that limited amount of chloride increased biomass and leaf area in *Nicotiana tabacum*, although the largest biomass variation was achieved through nitrate modulation [21]. The potential agronomical relevance of these effects, therefore, encourages new studies for sustainable crop management.

The impact of macro and micro-elements to increase yield and quality was studied in several aspects, and more detailed integrative investigations were recently conducted to determine the effect of the concurrent modulation of ions [22]. Since chloride may have beneficial effects especially in comparison with nitrate [14], in this work, we sought to examine the impact on basil of the simultaneous, opposite variation of nitrate and chlorine in nutrient solutions with the same ionic strength. Our hypothesis was to exploit the functional relations of the nitrate-chlorine modulation to sustainably increase basil quality while maintaining yield. To this aim, using a full factorial design, we evaluated changes in morpho-physiological, anatomical, chemical, and quality-related traits in basil in response to four different NO_3_^−^:Cl^−^ ratios in two consecutive harvests.

## 2. Materials and Methods

### 2.1. Plant Material, Growth Conditions and Experimental Design

At the three true leaves stage, sweet basil plantlets (*Ocimum basilicum* L. var. *basilicum*, large leaved Genovese type) were transferred to dark plastic pots with 1.3 L of a 2:1 mixture (*v*/*v*) of peat:perlite at the Experimental Station of the Department of Agricultural Sciences (University of Naples Federico II) in Bellizzi (SA), 43°31 N, 14°58 E (60 m a.s.l.). Transplant was carried out on April the 28th, 2016. Pots were in a greenhouse, and the mean air temperature during the experiments was 26 °C (min: 16 °C; max: 33 °C). The relative humidity was set at 57% during day and 80% during night. Plastic pots were on an aluminum bench and each experimental unit (plot) comprised ten plants. The first and the last two were treated as guard plants not to be analyzed. Plants were in single rows with a 23 plants per square meter density. The experimental design was a randomized complete-block design (RCBD), with three replicates per level of factor, for a total of 24 experimental units. As fixed factors we considered the NO_3_^−^:Cl^−^ ratio (R) in the nutrient solution (NS) (four levels) and the harvest (CT) of the leaves and stems (two levels). The four levels of factor R were named 80:20, 60:40, 40:60 and 20:80 according to the relative molar ratio of NO_3_^−^ and Cl^−^ in the nutrient solution. The NS was a modified Hoagland solution with the following composition: 1.5 mM phosphorus, 4.5 mM potassium, 6.5 mM calcium, 2.5 mM sulfur, 2.0 mM magnesium, 20 μM iron, 20 μM boron, 9 μM manganese, 1.6 μM zinc, 0.3 μM cupper, and 0.3 μM molybdenum. The electrical conductivity was 2.0 ± 0.1 dS m^−1^ and the pH was monitored and, when necessary, adjusted to be within the 6.0 ± 0.2 interval. We obtained the four NO_3_^−^:Cl^−^ ratios by using different amounts of Ca(NO_3_)_2_ and CaCl_2_. Specifically, nitrate concentration (expressed as NO_3_^−^ mM) and salt quantity (expressed as mg L^−1^ of Ca(NO_3_)_2_ and of CaCl_2_) in the NS were the following. Ratio 80:20: 9.6 mM, 867.1 and 133.2 mg L^−1^; 60:40: 7.2 mM, 650.3 and 266.4 mg L^−1^; 40:60: 4.8 mM, 433.5 and 399.6 mg L^−1^; 20:80: 2.4 mM, 216.8 and 532.8 mg L^−1^.

### 2.2. Harvest, Yield, Growth Analysis and Leaf Colorimetry

The sweet basil plants were harvested twice (47 and 75 days after transplanting, hereafter CT1 and CT2, respectively), when the plant height reached approximately 35 cm and before flowering. At each harvest date and for each experimental unit (i.e., replicate) six plants were collected. The above-ground plant was harvested and immediately weighed. Leaves were separated, weighed and the leaf area quantified with a LiCor 3100C area meter (LI-COR Biosciences, Lincoln, NE, USA). A sub-sample of the entire plant was dried in a forced-air oven at 70 °C for three days until constant weight to determine the shoot dry biomass. Dry matter percentage was also calculated using the following formula: DM = 100 × Dry weight/Fresh weight. On the center of the upper basil leaf surface 10 color measurements were performed with a Minolta Chroma Meter (CR-300, Minolta Camera Co. Ltd., Tokyo, Japan). The color space was measured as defined by the International Commission on Illumination, and color was expressed with three values: L* for the lightness from black (0) to white (100), a* from green (−) to red (+), and b* from blue (−) to yellow (+).

### 2.3. Quality Analysis Sampling

At both harvest dates, basil fresh samples (three replicates per treatment) were harvested, immediately frozen in liquid nitrogen, and stored at −80 °C before being lyophilized in a freeze-dryer Alpha 1-4 LSC Basic (M. Christ, Osterode, Germany) for further analysis (i.e., antioxidant activity, phytochemicals and volatile compounds). For the analysis of the mineral composition, the dry leaf material was milled with a Wiley Laboratory Mill model 4 to pass through an 841 µm sieve mesh.

### 2.4. Leaf Gas Exchange

Net photosynthetic carbon dioxide assimilation rate (A_CO2_) and transpiration (E) were determined just before the two harvests whit a portable gas exchange analyzer (LCA-4; ADC BioScientific Ltd., Hoddesdon, UK) equipped with a broadleaf chamber (cuvette window area: 6.25 cm^2^). The physiological measurements were carried out between 11.00 and 13.00 h on nine replicate plants per treatment. The climatic conditions (e.g., the photosynthetically active radiation, relative humidity, and carbon dioxide concentrations) were set at ambient temperature and the flow air rate was 400 mL s^−1^. Intrinsic water use efficiency was calculated as the ratio between A_CO2_ and E.

### 2.5. Nitrate and Mineral Analysis

One gram of basil ground material was used for the determination of total nitrogen (N) using the Kjeldahl method [23]. Ground material (0.25 g) was analyzed by ion chromatography (ICS-3000, Dionex, Sunnyvale, CA, USA) to determine the mineral content (P, K, S and Cl) as described [24]. The results were expressed as g kg^−1^ dry weight (dw), whereas nitrate was expressed as mg kg^−1^ fresh weight (fw), according to each sample dry matter content.

### 2.6. Total Chlorophyll and Total Ascorbic Acid Measurement

Total chlorophyll and total ascorbic acid (TAA) were analyzed on the fresh basil material. Total chlorophyll content (chlorophyll a and b) and TAA were determined as reported [25,26] using a Hach DR 4000 (Hach Co., Loveland, CO, USA) spectrophotometer. Total chlorophyll and TAA were expressed in µg g^−1^ and in mg AA 100 g^−1^ fw, respectively.

### 2.7. Antioxidant Activity and Total Phenols Analyses

The lipophilic antioxidant activity (LAA) and the total phenols (TP) content were analyzed on freeze-dried leaf samples using UV–Vis spectrophotometric approached [27,28]. Absorbance was measured at 505 nm for LAA and 525 nm for TP. LAA and TP were expressed in mmol eq. trolox 100 g^−1^ and mg eq. gallic acid g^−1^, respectively.

### 2.8. Analysis of Volatile Compounds

Flavor compounds (e.g., volatiles and semi-volatiles, C3–C20) were analyzed using the solid-phase microextraction (SMPE) sampling technique coupled with gas chromatography/mass spectrometry (GC/MS). Compounds were analyzed using three biological replicates. An SPME fiber assembly coated with Divinylbenzene/Carboxen/Polydimethylsiloxane (DVB/CAR/PDMS; film thickness: 50/30 µm) (Supelco, Bellefonte, PA, USA) was employed for the extraction of the analytes. Freeze-dried powdered leaves (2 g) were introduced into a 20 mL vial of headspace. The SPME fiber was exposed in the headspace for 20 min at 45 °C and then, introduced into the GC injector. Analytes desorption was carried out at 250 °C for 5 min. GC/MS was performed in a6890N Network Gas Chromatograph (Agilent Technologies, Santa Clara, CA, USA) equipped with a 5973 Mass Detector System (MDS) (Agilent Technologies). Separation was carried out on a 30 m × 0.250 mm capillary column coated with 0.25 µm of 5% diphenyl −95% dimethylpolysiloxane (HP5MS J&W Scientific, Folsom, CA, USA), directly inserted into the ion source of the MDS. After column conditioning (50 °C for 2 min), oven temperature was ramped with a 10 °C per minute speed up to 150 °C and then, with a 15 °C per minute speed up to 300 °C, which was held for 10 min. The spitless injection and ion source temperatures were 250 and 230 °C, respectively. Helium (99.999%) was used as the carrier gas at a flow rate of 1 mL per minute. The electron ionization energy was 70 eV. The scanned mass range was 40–450 atomic mass units (in full-scan mode). Volatile compounds were identified considering their mass spectra and retention indices using the NIST Atomic Spectra Database version 1.6 libraries, with a range of similarity values of 85–100%. Relative quantification was performed on the basis of the peak area.

### 2.9. Leaf Anatomical Traits

Leaf sampling for the anatomical analyses was performed at the second harvest stage (CT2) on six replicate plants per treatment. The lamina of fully expanded leaves was fixed in a solution made of 38% formaldehyde, glacial acetic acid, 50% ethanol (5:5:90 by volume). For the analysis of epidermal traits, epidermal strips were peeled off from the abaxial and the adaxial lamina surfaces, flattened, and mounted with distilled water on microscope slides. Peels were observed under an epi-fluorescence microscope (BX60; Olympus, Hamburg, Germany) set to detect, with different emissions, stomata and glandular trichomes (mercury lamp, band-pass filter of 330–385 nm, dichromatic mirror of 400 nm and above, and a barrier filter of 420 nm and above). Collected images (Camedia C4040; Olympus) were analyzed through the software program AnalySIS 3.2 (Olympus) to quantify stomata frequency (SF) and size (area of each couple of guard cells – GCA, guard cells area). SF was calculated in three regions per peel, while GCA was measured in 20 stomata per peel. For the analyses of mesophyll traits, leaf lamina samples were divided into 5 × 5 mm pieces, which were dehydrated in a graded ethanol series (up to 95%), infiltrated and embedded in the acrylic resin JB4 (Polysciences, Hirschberg, Germany). Cross-sections (5-µm thick) were cut through a rotary microtome, stained with 0.5% Toludine blue in water [29], mounted with Canadian balsam and observed under a light microscope (BX60, Olympus). Digital images were captured and analyzed as reported above to measure: the thickness and area of palisade and spongy cells, and the density of spongy parenchyma in terms of quantity of intercellular spaces (IS, expressed as the percentage of tissue occupied by air spaces over a given surface) [30]. All these traits were measured in six positions along the lamina, avoiding veins.

### 2.10. Statistics

The statistical analyses were carried out using a two-way between-subject analysis of variance (ANOVA), followed by post-hoc analysis with the Duncan’s multiple range-test (α < 0.05). The fixed factors were the NO_3_^−^:Cl^−^ ratio (R) in the nutrient solution, and the harvest (CT) of the leaves and stems. Differences between cuts were evaluated with an independent Student’s t-test. For anatomical traits, a one-way ANOVA was performed followed by post-hoc analysis with the Duncan’s multiple range-test (α < 0.05). The statistical analysis was carried out with the Statistical Package for Social Sciences (SPSS) software version 20 (IBM Corp., Armonk, NY, USA). Principal Component Analysis and correlation analysis were performed and visualized in R [31].

## 3. Results

### 3.1. Multivariate Analysis of the Basil Response to the NO_3_^−^:Cl^−^ Ratio and Cut

**Figure 1:**
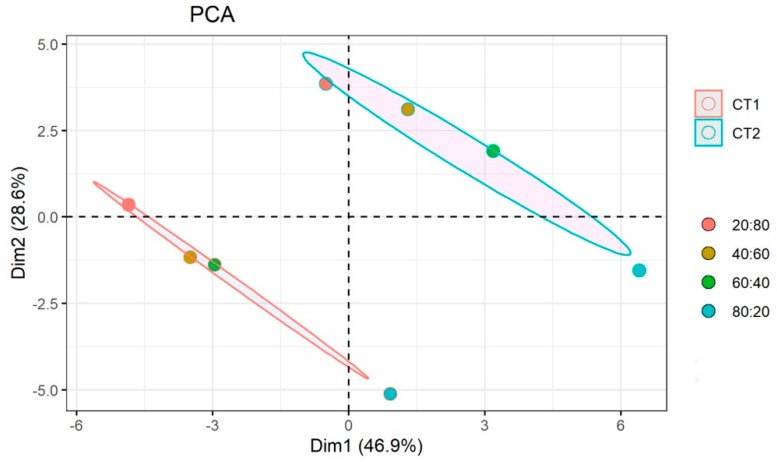
Principal Component Analysis of the basil response. Each condition is represented by a circle, colored according to the different NO_3_^−^:Cl^−^ ratios (20:80, 40:60, 60:40, 80:20). For each cut (CT1 and CT2), the plot displays in different colors the confidence ellipse around the mean point (not shown). The confidence level was 0.95. Color legend is reported on the right-hand side.

The effects of the different nutrient regimes (R) and number of cuts (CT) on basil were evaluated considering agronomic, physiological, biochemical, and quality-related traits. To visualize the resemblance between biological samples and conditions, we first performed a multivariate analysis on the continuous variables (*n* = 29). Principal component analysis (PCA) provided an overview of the plant response to the different nutrient solutions and harvests. The first two principal components (PCs) explained 75.5% of the total variance (Supplementary Figure S1) and were used to graphically represent samples’ relations (Figure 1).

The experimental conditions could be grouped in two clusters well associated with the CT factor. Moreover, for both harvests, the samples were progressively separated along the two axes according to the variation of the nutrient solution (NS), consistent with a gradual response of the plant. PC1, which accounts for 46.9% of the variance, mainly separated samples according to the NS. Finally, the PCA analysis indicated that the effect on all the variables under investigation was larger at the second cut, in particular as regards PC2. Overall, the data indicated a progressive basil response to the nutrient solution with a separation among experimental conditions significantly affected by the CT factor.

### 3.2. Effect on Plant Growth and Water Balance

Both tested factors (R and CT) and in some instances, their interaction (R × CT) altered basil fresh yield and growth response (Table 1).

**Figure 2:**
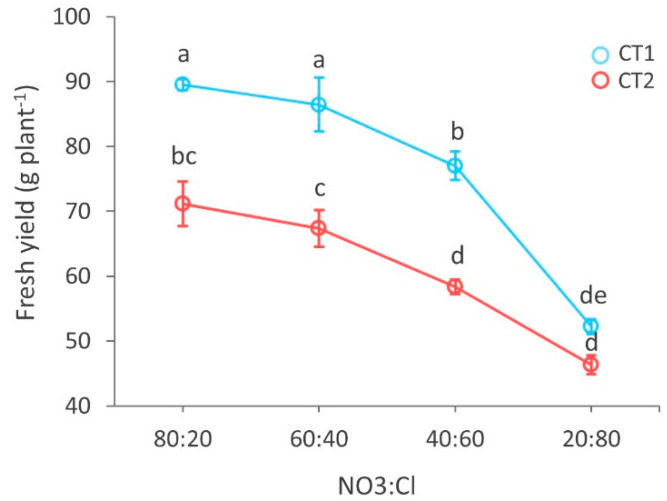
Main effect of the NO_3_^−^:Cl^−^ ratio in the nutrient solution in the two harvests (CT1, azure; CT2, salmon) on fresh yield of basil. Different letters indicate significant differences according to Duncan’s test (*p* = 0.05). All data are expressed as mean standard error, *n* = 3.

Specifically, the NS significantly affected leaf number and area, as well as fresh yield, dry shoot biomass (leaves and stems) and dry matter percentage. For example, in both CT1 and CT2, fresh yield decreased with increasing chlorine concentrations in the NS, and the highest crop productivity was recorded at the 80:20 and 60:40 treatments (Figure 2). Moreover, basil plants supplemented with the two highest nitrate concentrations (i.e., 80:20 and 60:40) exhibited the highest biomass and number of leaves. The reduction of nitrates in the NS associated with a decline of all the parameters except for the percentage of dry matter, which increased (~25%) only at the higher chlorine concentration (Table 1). Considering the dry shoot biomass, this relative increase is due to a strong decrease in the water content. The other parameters also show the highest relative difference from the 40:60 R to the 20:80 R.

The main effect of the CT was more complex, because at CT2 an increase in leaf number associated with a decrease of the leaf area, resulting in an unchanged above-ground biomass (i.e., leaves and stem). At CT2, the percentage of dry matter also increased compared to first cut (CT1), mainly because of a lower water content. The percentage of dry matter was significantly altered also by the R × CT interaction, indicating that the effect of the nutrient ratios on the water content changed in the two growing phases. The number of leaves also displayed a significant non-antagonist R × CT interaction, and at lower nitrate contents, their reduction was higher at CT2 than at CT1.

### 3.3. Effect on Total Chlorophyll Content and Photosynthesis

The consequence of different nutrient solutions and cuts on basil was also evaluated at physiological level (Table 2).

Interestingly, the reduction of NO_3_^−^ concentration in the NS was not accompanied by a significant reduction of the total chlorophyll content in leaves. Chlorophyll was reduced at the second cut, and the NS and factors’ interaction did not have a significant effect. On the other hand, the net CO_2_ assimilation was influenced by both factors and their interaction. Specifically, lower NO_3_^−^:Cl^−^ ratios progressively decreased the net photosynthetic rate. At the 20:80, its value was approximately 60% of the 80:20 treatment. This reduction was significantly higher for leaves growing after the first harvest. The NO_3_^−^:Cl^−^ ratio and the cut significantly altered also the transpiration rate. However, a linear response to the NS was not evident, while the CT almost doubled this parameter. A progressive response to the NS was not present for the instantaneous intrinsic water use efficiency (iWUE), which was higher at the 80:20 R compared to the other nutrient ratios. At the second cut, iWUE was markedly lower than at the first one, but there was not a significant interaction with the R factor (Table 2).

### 3.4. Effect on the Ionic Accumulation in Leaves

We also analyzed the mineral content of the leaves, namely nitrate, nitrogen, chlorine, phosphorous, potassium and sulfur concentration (Table 3).

**Figure 3:**
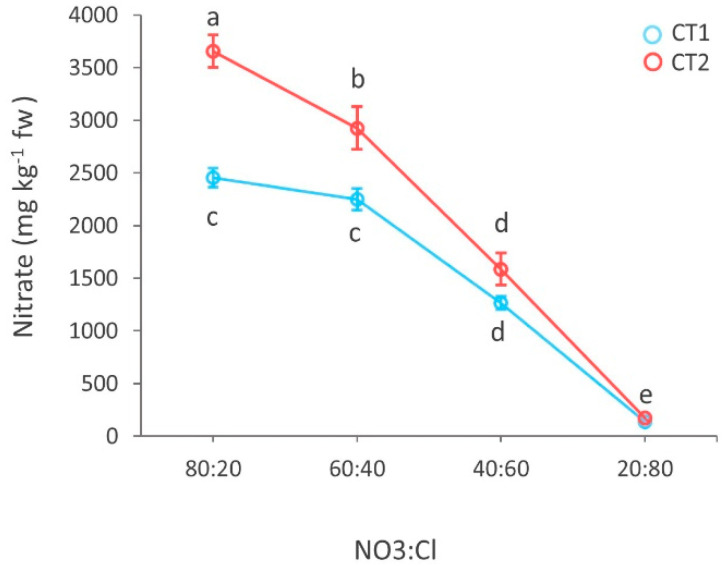
Main effect of the NO_3_^−^:Cl^−^ ratios in the nutrient solution in the two harvest on nitrate content of basil (CT1: azure; CT2: salmon). Different letters indicate significant differences according to Duncan’s test (*p* = 0.05). All data are expressed as mean standard error, *n* = 3.

Nitrate content in leaves decreased with increasing Cl^−^ in the nutrient solution, with more pronounced effects at CT2. Specifically, nitrate content decreased linearly with Cl^−^ availability at CT2, whereas for 80:20 and 60:40, the nitrate content at CT1 remained unchanged (Figure 3). The concentration of nitrogen well associated with the different NO_3_^−^:Cl^−^ ratios and did not differ between cuts. The nitrogen uptake efficiency (i.e., the ratio between nitrogen content in leaves and in the nutrient solution) increased linearly according to rising Cl amounts. The NS significantly affected chlorine concentration in leaves, which progressively doubled with decreasing NO_3_^−^:Cl^−^ R. Chlorine concentration was higher at the second cut and was also positively affected by the R × CT interaction. Factors’ interaction was not significant for the other minerals under investigation. P and K concentration was differentially affected by either the NS or the CT factor while the S concentration did not display a significant variation.

### 3.5. Effect on Leaf Color and Antioxidant Capacity

The effect of the R and CT factors on basil leaves was analyzed identifying color differences as well as some biochemical features that define product quality (Table 4). Leaf color was evaluated using the L*a*b* coordinates. These variables were statistically affected by R and CT but not by their interaction. Specifically, changes of the NO_3_^−^:Cl^−^ ratio determined a significant variation for the L* component (i.e., lightness) only at the 20:80 R. The parameters a* and b*, which express the chromaticity of the leaves, were affected principally by the cut. Overall, the lighter coloring (highest yellowness (+b*) and maximum lightness (L*)) was recorded in basil leaves at 20:80, and this effect was more marked at CT2. Although statistically significant, this phenotype is little perceived by the human eye, and evident only by pairwise comparisons. The lipophilic antioxidant capacity (LAA) was significantly reduced at the CT2, similarly to the TAA. However, TAA concentration almost doubled at the 20:80 R compared to the 80:20 R, while the NS had a negligible influence on LAA. Total phenols (TP) also progressively increased with lowering R but differences between cuts were not significant. TP and TAA positively correlated with the dry matter and the chlorine concentration in leaves.

### 3.6. Effect on Volatiles

Finally, we evaluated the effects on main components of the basil aroma and fragrance (Table 5). Overall, the data indicated that the nutrient solution R, the CT, and their interaction specifically altered the aromatic profile of the leaves. For instance, the relative number of metabolites connected with the jasmonic acid pathway was differentially affected. Specifically, while the secondary alcohol 1-octen-3-ol did not display variation, the leaf aldehyde 2-hexenal (E)- was significantly higher in leaves growing after the first harvest. A similar trend was also observed for β-ocimene, a monoterpene associated with plant stress response (Table 5). Other volatiles were not affected by the CT factor. Compared to the CT factor, the effect of the NS was larger, as the percentage of four compounds displayed a statistical variation. The R × CT interaction strongly affected eugenol. This plant stress-elicited compound, which ultimately derives from the L-tyrosine metabolism, had the highest concentration at the 60:40 and the lowest at 80:20. Among the tested compounds, β-myrcene was the only monoterpene that affected by the nutrient solution and its interaction with the harvest.

### 3.7. Correlation Analysis

To discover and visualize the pairwise relationships between variables, we built a correlogram for each cut (Supplementary Figure S2). Overall, pairwise correlations were more evident for leaves of the second cut. As expected, the nitrate content in the leaves was the variable showing the largest number of statistically significant positive correlations. The correlation analysis confirmed that nitrogen and chlorine concentration in leaves were the variables that most associated with others, with N (resp. Cl) displaying substantially positive (resp. negative) correlations. The correlation of nitrogen and chlorine content in leaves with agronomic and physiological variables was stronger at the second cut. The correlation analysis also showed the strong negative correlation between N and total phenols, while the hydrophilic antioxidants capacity well correlated with the physiological variables, especially the photosynthetic rate. Finally, the component of the aromatic profile little correlates with the other variables, apart from hexanal that in different instances, negatively correlated with the physiological parameters under investigation.

### 3.8. Effect on Leaf Anatomical Traits

**Figure 4:**
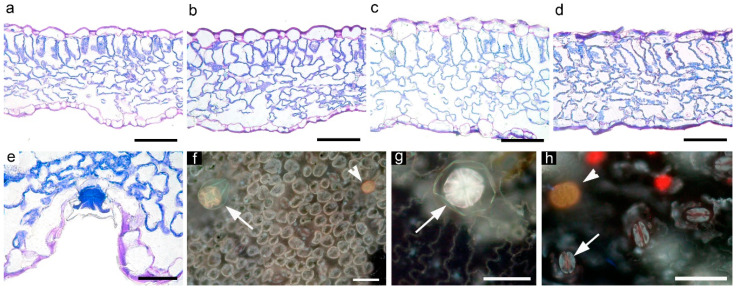
Transmitted light (**a**–**e**) and epi-fluorescence (**f**–**h**) microscopy views of cross sections (**a**–**e**) and epidermal peels (**f**–**h**) of basil leaves subjected to the four different NO_3_^−^:Cl^−^ ratios in the nutrient solution (**a**, 80:20; **b**, 40:60; **c**, 60:40; **d**, 20:80). Details of glandular trichomes and stomata: (**e**,**f**,**g**) (long arrows) show peltate glands; (**f**,**h**) (short arrows) show capitate glands; long arrow in (**h**) shows a stomata. Scale bars are 100 mm in (**a**–**d**), 50 mm in (**e**–**h**).

The excessive accumulation of chloride may lead to anatomical disarrangements in leaves. We used chloride concentrations that are not predicted to rise an acute plant response (e.g., chlorosis or burned appearance) yet, to verify possible effects of prolonged exposure to chloride, we analyzed the leaf anatomy at the end of our experiments. Microscopy observations of the lamina cross sections of the dorsiventral basil leaves showed that increasing Cl^−^ in the NS was responsible for the occurrence of alterations, slightly visible at 40:60 R and more marked at 20:80 R. Specifically, leaves of plants treated with the two highest Cl^−^ solutions showed signs of cell shrinkage, loss of turgidity of the mesophyll cells, and a less uniform, often broken cuticle (Figure 4a–d). Stomata, capitate and peltate glandular trichomes (Figure 4e–h) were evident on both abaxial and adaxial epidermis, with glandular trichomes more frequent on the adaxial lamina surface.

On the abaxial epidermis, stomata frequency was not significantly influenced by the NS, while stomata size was significantly reduced at 20:80 (Table 6). On the adaxial epidermis, both stomata and glands frequency were progressively reduced according to the decrease in NO_3_^−^:Cl^−^ ratio in the NT, while stomata size tended to decrease with the two highest Cl^−^ solutions (Table 6). The highest lamina thickness was at 40:60 R, mainly due to thicker palisade parenchyma (Table 7). Spongy parenchyma tissue was significantly higher at 60:40 and 40:60 compared to the other treatments. Changes in the tissues thickness followed the changes in palisade and spongy cell size. Finally, intercellular spaces followed a decreasing trend according to the increase in Cl^−^ in the NT, with minimum values at 20:80 (Table 7).

## 4. Discussion

In this work, we demonstrated the broad range of alterations in basil caused by the variation of two antagonist anions (nitrate and chloride) in four nutritional formulations that have the same anion concentration. We also compared leaves from two harvests, considering that basil is harvested at least twice in professional horticulture.

Crops are typically supplied with ample inorganic nutrients and it is well demonstrated that nitrogen fertilization is a key determinant of plant growth. Accordingly, the highest yield was obtained at the highest nitrate concentration (i.e., the 80:20). Moreover, the overall yield was higher at the first cut [32,33]. Nonetheless, it is interesting that yield did not significantly decrease with the 60:40 ratio. A large yield reduction was evident for the 20:80 and only at this ratio, the yield at CT2 was not statistically lower than at CT1. Yield differences can be explained by the concomitant, specific action of the two anions. As expected for nitrogen fertilization, yield and dry biomass correlated well and likewise, plants with higher yield had larger leaves. The dry biomass did not show differences between cuts, while the percentage of dry matter was always higher at CT2, indicating a predominant role of the water content in yield differences. Water content was visibly reduced at high chlorine concentration and the strong yield reduction at 20:80 associated with a strong increase in the percentage of dry matter. The reduction of leaf lamina according to the decrease in the nitrogen/chloride ratio in the nutrient solution, was accompanied by an increase in the leaf lamina thickness up to 40:60. The trend of increasing thickness was inverted at 20:80, where the mesophyll showed signs of stress (e.g., severe cell shrinkage) that are consistent with the reduced water content. It is worth adding that in our experimental system, different amounts of chlorine were supplied with solutions with the same osmotic potential, therefore not affecting water movement. For instance, the constant amount of sulfur in leaves is consistent with the lack antagonism with the sulfate anion in the NS [34]. Similarly, a gradual antagonist effect of chlorine on phosphate accumulation was not evident, and phosphate content in leaves was reduced only at the 20:80 ratio [16,18]. In addition, the reduction of nitrate in the NS (and in leaves) did not cause a significant decrease in chlorophyll, pointing towards a compensatory effect of lower concentrations of chlorine, which may be present also for gas exchange parameters. For instance, chlorophyll content was significantly reduced at the second cut and net photosynthetic carbon dioxide assimilation rate only mildly decreased with lower nitrogen supplies. Being present in plants with a smaller leaf surface, the lower assimilation rate per leaf area should result in a large effect on the whole plant photosynthetic activity. Moreover, the reduction in A_CO2_, along with an unchanged or increase in evapotranspiration, according to the increase in higher chlorine concentration can be explained by a coordination of leaf structural traits [35,36]. At 40:60, the occurrence of thicker leaves (due to larger cells) coupled with reduced intercellular spaces explains the higher evapotranspiration due to the maintenance of high the leaf hydraulic conductance (Kleaf), but reduced photosynthesis due to lower volume available for CO_2_ exchange in comparison with the other conditions. Many studies indicated a negative effect of low nitrogen supply on intrinsic water use efficiency [37]. iWUE, in both cuts, reduced only from 80:20 to 60:40, suggesting that Cl allows maintaining the same iWUE regardless of a mild increase of transpiration and a reduced biomass. In addition, the adjustment of stomata traits at the adaxial lamina surface supports the induction of a leaf response towards the quicker control of gas exchange thanks to a reduction in the frequency and size of stomata at 20:80 and 40:60. For instance, smaller stomata allow a better and faster stomatal response [38].

Plants treated with the highest amount of nitrate (resp. chloride) showed the highest leaf content of total nitrogen and nitrate (resp. chlorine). Only chlorine accumulated in higher amount at the second cut, mostly because nitrate is continuously metabolized to sustain the growth after the first harvest, unlike chlorine. The maximum chlorine accumulation in leaves was 1.6 mmol per g dw, a value in the range of a macronutrient. At the 20:80 ratio, the Cl concentration in leaves exceeded the maximum of the conventional range in glycophytes (20 g kg^−1^ dw) [19]. This amount is insufficient to cause visible symptoms of toxicity [21] (we detected only at microscopic level evidence of anatomical perturbation). For instance, in sweet basil salt stress-induced morphological effects are usually visible starting from a 25 mM NaCl concentration [39]. A linear response of the mineral composition to the nutrient solution was not evident for all R. The sharp decrease in nitrate and total nitrogen at the 20:80 R, along with the increased efficiency in nitrogen uptake and use at increasing Cl concentration in the NS, suggest that nutrient availability or anion-anion competition in uptake and mobilization are not the only factors that can explain mineral composition of the leaves. In leaves, nitrogen is mainly present in photosynthetic enzymes [40,41], and it is well established that total nitrogen content strongly correlates with nitrate and the net photosynthetic rate. In our system, the latter correlation was mild and present mainly for leaves of the second cut. As also recorded for iWUE, this can be explained considering a compensatory effect of chlorine that allows Cl to sustain CO_2_ assimilation rate only when present in leaves in the range of a macronutrient (mmol) but not in high amount (roughly, below 5 mmol g dw) [21,42].

The nutrient solution also affected basil nutritional properties. As expected, the concentration of total ascorbic acid was inversely correlated with nitrate fertilization [43]. For leafy products, this is typically justified considering the strong positive correlation between nitrogen and plant biomass. Moreover, also a mild supplement of NaCl is reported to increase the ascorbic acid concentration [8]. For instance, the addition of 40 mM NaCl in the NS increased the TAA and TP content in soilless cultivated basil [44]. Similarly, higher polyphenolic contents were observed with reducing nitrates in the NS, consistent with the C/N balance theory [45]. The lipid-soluble antioxidants content was the quality-related component that little correlated with other variables, including chlorophyll and biomass, and was only affected by the CT factor. Variation of carotenoids content in leaves is usually described in relation to stress, and in sweet basil NaCl salinity induced an increase in carotenoids concentrations of the leaf tissue [39].

The effect on the volatile compounds was complex. In comparison with the other variables, the aromatic profile was the least affected by the two factors (CT: two compounds out of eight; R: four), and their interaction (two). The frequency of secretory glandular trichomes was reduced according to the decrease in nitrogen supply already at 60:20. NS composition likely affected not only the differentiation of such trichomes at leaf surface, but also their capacity to synthesize and accumulate phenylpropenes. It was previously demonstrated that the reduction of the frequency of trichomes that actively produce volatile oils was not necessarily accompanied by the decrease in the major aroma volatiles analyzed [4]. Among the major components that are significant for basil aroma and for its antioxidant properties, eugenol was affected by the two factors and their interaction. However, a linear correlation with the variation of the NS ratios was not present. Nitrogen application contributed to a decrease in the contents of volatiles such as linalool, the major component of the European-basil oil [46]. On the other hand, it has also been proposed that nitrogen application can increase volatile emission through an expansion of the leaf surface [47]. In our study, the analyzed chemical compounds little correlated with the morphological parameters and in a few cases, such as for hexanal, negatively correlated with yield and leaf area, as well as the photosynthetic rate. A compensatory effect of the two anions cannot be excluded however, very little is known on the effect of chlorine on the aromatic compounds, besides a positive effect of the NaCl stress on essential oil production [39].

## 5. Conclusions

Our investigation revealed the effect of the simultaneous variation of two antagonist anions in basil. Almost all the recorded parameters were affected and overall, the effect was multifaceted and often consistent with the different roles of nitrate and chlorine in fertilization. Very briefly, the data indicated that the concomitant reduction of nitrate and increase of chloride in the NS induces basil to produce a higher number of leaves, with a smaller area and a reduced mesophyll density and glands frequency. Even at the maximum employed concentration of chlorine, the overlap with the salt stress-induced basil phenotype was limited [39]. The technically simple moderate increase of chlorine in the NS can provide compensatory advantages such an increase iWUE without reducing the photosynthetic efficiency, possibly counteracting the expected reduction of plant parameters strongly related to nitrogen fertilization [14]. Lower inputs of nitrate are welcome in agriculture to reduce production and environmental costs. It is well known that the main source of nitrogen pollution and related ecological damage comes from N-based agricultural fertilizers. Taking as reference the highest nitrate supply, the 60:40 ratio provided an acceptable growth and appearance, with no signs of structural stress even at microscopy level. In addition, considering that the nutraceutical and pharmacological properties of basil resides in its secondary metabolites (and especially its antioxidant capacity), the 60:40 ratio also offered some quality-related added values. Finally, our work also demonstrated the ample differences in plant response to the NS that exist between two consecutive harvests. The evidence provided made it possible to define more sustainable growing conditions and opened the door to further improvement in basil quality by fine-tuning the nutrient solution.

## Figures and Tables

**Table 1:** Analysis of variance and mean comparisons for growth parameters and biomass production of greenhouse basil cultivated under four NO_3_^−^:Cl^−^ ratios (R) in the nutrient solution and harvested in two successive cuts (CT).

Source of Variance	Leaf Number	Leaf Area	Dry Shoot Biomass	Dry Matter
	(n plant^−1^)	(cm^2^ plant^−1^)	(g plant^−1^)	(%)
NO_3_^−^:Cl^−^ ratio (R)				
80:20	100.4 ± 9.48 a	1917 ± 137 a	9.54 ± 0.34 a	9.11 ± 0.66 b
60:40	94.7 ± 8.92 a	1787 ± 133 b	8.99 ± 0.37 a	9.06 ± 0.58 b
40:60	86.6 ± 4.91 b	1659 ± 131 c	8.18 ± 0.28 b	9.35 ± 0.56 b
20:80	75.2 ± 4.97 c	1249 ± 71 d	7.03 ± 0.14 c	11.74 ± 1.08 a
	***	***	***	***
Cuts (CT)				
CT1	74.1 ± 2.20	1902 ± 97	8.57 ± 0.36	8.26 ± 0.22
CT2	104.4 ± 4.42	1404 ± 64	8.30 ± 0.33	11.37 ± 0.50
t-value	***	***	ns	***
R × CT				
80:20 × CT1	79.7 ± 4.28 c	2211 ± 46	9.52 ± 0.05	7.72 ± 0.12 d
60:40 × CT1	76.3 ± 3.44 c	2055 ± 117	9.29 ± 0.69	7.78 ± 0.05 d
40:60 × CT1	76.2 ± 2.12 c	1941 ± 74	8.60 ± 0.42	8.11 ± 0.09 d
20:80 × CT1	64.3 ± 1.83 d	1402 ± 32	6.86 ± 0.04	9.42 ± 0.24 c
80:20 × CT2	121.1 ± 1.99 a	1622 ± 75	9.56 ± 0.75	10.51 ± 0.44 b
60:40 × CT2	113.2 ± 6.70 a	1519 ± 54	8.69 ± 0.31	10.34 ± 0.18 b
40:60 × CT2	97.1 ± 2.67 b	1377 ± 36	7.76 ± 0.23	10.59 ± 0.16 b
20:80 × CT2	86.1 ± 1.48 c	1095 ± 28	7.20 ± 0.26	14.06 ± 0.60 a
	*	ns	ns	**

Asterisks indicate a statistically significant difference (***: *p* < 0.001; **: *p* < 0.01; *: *p* < 0.05; ns: non-significant, *p* ≥ 0.05). For each source of variance, different letters within each column indicate statistically different groups according to the Duncan’s post-hoc test (*p* = 0.05). The factor “Cut” was analyzed using an independent Student’s t-test. Data are mean values ± standard error, *n* = 3.

**Table 2:** Analysis of variance and mean comparisons for chlorophyll content and leaf gas exchange parameters of greenhouse basil cultivated under four NO_3_^−^:Cl^−^ ratios in the nutrient solution and harvested in two successive cuts.

Source of Variance	Chlorophyll	A_CO2_	E	iWUE
	(µg/g fw)	(μmol CO_2_ m^−2^ s^−1^)	(mol H_2_O m^−2^ s^−1^)	(μmol CO_2_ mol^−1^ H_2_O)
NO_3_^−^:Cl^−^ ratio (R)				
80:20	252.2 ± 20.9	13.35 ± 0.74 a	2.63 ± 0.37 b	5.77 ± 1.02 a
60:40	227.3 ± 13.8	10.05 ± 1.54 b	3.58 ± 0.60 a	3.67 ± 1.09 b
40:60	216.0 ± 17.3	8.95 ± 1.75 bc	3.19 ± 0.42 ab	3.41 ± 0.96 b
20:80	202.8 ± 21.0	8.04 ± 1.76 c	3.32 ± 0.48 a	3.14 ± 1.01 b
	ns	***	*	***
Cuts (CT)				
CT1	255.2 ± 10.0	13.20 ± 0.47	2.20 ± 0.09	6.18 ± 0.42
CT2	194.0 ± 10.0	6.99 ± 0.91	4.16 ± 0.21	1.81 ± 0.32
t-value	***	***	***	***
R × CT				
80:20 × CT1	272.7 ± 38.9	14.85 ± 0.39 a	1.89 ± 0.19	7.97 ± 0.55
60:40 × CT1	248.4 ± 16.2	13.22 ± 1.35 ab	2.29 ± 0.20	5.92 ± 0.94
40:60 × CT1	250.8 ± 13.7	12.80 ± 0.61 b	2.34 ± 0.11	5.51 ± 0.46
20:80 × CT1	248.9 ± 6.6	11.95 ± 0.48 b	2.28 ± 0.20	5.33 ± 0.52
80:20 × CT2	231.8 ± 15.9	11.85 ± 0.58 b	3.36 ± 0.32	3.56 ± 0.23
60:40 × CT2	206.2 ± 15.5	6.88 ± 0.14 c	4.87 ± 0.32	1.43 ± 0.11
40:60 × CT2	181.1 ± 9.5	5.11 ± 0.39 cd	4.03 ± 0.41	1.31 ± 0.24
20:80 × CT2	156.8 ± 6.1	4.12 ± 0.12 d	4.36 ± 0.05	0.95 ± 0.04
	ns	**	ns	ns

Asterisks indicate a statistically significant difference (***: *p* < 0.001; **: *p* < 0.01; *: *p* < 0.05; ns: non-significant, *p* ≥ 0.05). For each source of variance, different letters within each column indicate statistically different groups according to the Duncan’s post-hoc test (*p* = 0.05). The factor “Cut” was analyzed using an independent Student’s t-test. Data are mean values ± standard error, *n* = 3.

**Table 3:** Analysis of variance and mean comparisons for mineral composition of greenhouse basil cultivated under four NO_3_^−^:Cl^−^ ratios (R) in the nutrient solution and harvested in two successive cuts (CT).

Source of Variance	Total N	Cl	P	K	S
	(g kg^−1^ dw)	(g kg^−1^ dw)	(g kg^−1^ dw)	(g kg^−1^ dw)	(g kg^−1^ dw)
NO_3_^−^:Cl^−^ ratio (R)					
80:20	42.70 ± 0.47 a	5.00 ± 0.89 d	9.41 ± 1.23 a	50.68 ± 6.26 b	1.23 ± 0.16
60:40	39.50 ± 0.68 b	11.66 ± 1.51 c	10.15 ± 0.57 a	48.79 ± 5.61 b	1.22 ± 0.08
40:60	36.01 ± 0.36 c	21.55 ± 4.41 b	7.92 ± 0.49 a	57.40 ± 5.55 a	1.26 ± 0.11
20:80	25.29 ± 0.94 d	40.89 ± 6.16 a	4.72 ± 0.50 b	44.32 ± 6.11 b	1.29 ± 0.21
	***	***	***	**	ns
Cuts (CT)					
CT1	35.74 ± 1.75	13.01 ± 2.78	7.81 ± 0.98	38.80 ± 2.60	1.38 ± 0.12
CT2	36.01 ± 2.26	26.54 ± 5.57	8.29 ± 0.59	61.80 ± 2.18	1.12 ± 0.05
t-value	ns	*	ns	***	ns
R × CT					
80:20 × CT1	41.69 ± 0.16 ab	3.90 ± 1.61 e	9.96 ± 2.45	40.29 ± 6.44	1.35 ± 0.29
60:40 × CT1	38.41 ± 0.90 cd	8.37 ± 0.72 de	10.27 ± 1.21	36.85 ± 1.01	1.30 ± 0.15
40:60 × CT1	36.31 ± 0.75 de	12.44 ± 2.76 cd	7.07 ± 0.58	46.11 ± 4.57	1.28 ± 0.21
20:80 × CT1	26.55 ± 1.60 f	27.34 ± 2.05 b	3.94 ± 0.64	31.93 ± 5.61	1.58 ± 0.38
80:20 × CT2	43.72 ± 0.21 a	6.11 ± 0.39 e	8.87 ± 1.16	61.07 ± 6.81	1.10 ± 0.16
60:40 × CT2	40.58 ± 0.57 bc	14.95 ± 0.34 c	10.04 ± 0.36	60.72 ± 3.74	1.15 ± 0.03
40:60 × CT2	35.72 ± 0.09 e	30.66 ± 2.56 b	8.76 ± 0.39	68.69 ± 2.36	1.24 ± 0.12
20:80 × CT2	24.03 ± 0.55 g	54.44 ± 1.25 a	5.49 ± 0.49	56.71 ± 1.17	0.99 ± 0.03
	*	***	ns	ns	ns

Asterisks indicate a statistically significant difference (***: *p* < 0.001; **: *p* < 0.01; *: *p* < 0.05; ns: non-significant, *p* ≥ 0.05). For each source of variance, different letters within each column indicate statistically different groups according to the Duncan’s post-hoc test (*p* = 0.05). The factor “Cut” was analyzed using an independent Student’s t-test. Data are mean values ± standard error, *n* = 3.

**Table 4:** Analysis of variance and mean comparisons for Hunter color parameters L * (brightness), a * (-a * = green) and b * (+b * = yellow), lipophilic and hydrophilic antioxidant activities (LAA and HAA), total phenols and total ascorbic acid (TAA) of greenhouse basil cultivated under four NO_3_^−^:Cl^−^ ratios (R) in the nutrient solution and harvested in two sequential cuts (CT).

Source of Variance	L *	a *	b *	LAA	Total Phenols	TAA
				(mmol Trolox 100 g^−1^ dw)	(mg gallic ac. 100 g^−1^ dw)	(mg 100 g^−1^ fw)
NO_3_^−^:Cl^−^ ratio (R)						
80:20	40.81 ± 0.56 b	−12.64 ± 1.83 a	19.66 ± 1.05 b	11.85 ± 0.57	62.50 ± 2.07 c	32.42 ± 1.79 c
60:40	42.04 ± 0.48 b	−13.71 ± 2.01 ab	21.70 ± 1.40 ab	13.55 ± 1.55	70.75 ± 3.38 c	40.34 ± 6.79 bc
40:60	41.69 ± 0.70 b	−13.18 ± 1.84 ab	20.74 ± 1.41 b	13.24 ± 1.90	99.50 ± 4.26 b	47.43 ± 8.08 b
20:80	44.54 ± 0.95 a	−14.38 ± 2.04 b	24.50 ± 1.21 a	12.79 ± 0.91	125.95 ± 11.1 a	61.47 ± 5.85 a
	***	*	*	ns	***	***
Cuts (CT)						
CT1	41.25 ± 0.40	−9.23 ± 0.25	19.56 ± 0.81	15.21 ± 0.72	98.59 ± 10.3	53.97 ± 4.32
CT2	43.29 ± 0.66	−17.72 ± 0.35	23.74 ± 0.78	10.51 ± 0.43	80.77 ± 5.45	36.86 ± 4.75
t-value	*	***	***	***	ns	*
R × CT						
80:20 × CT1	40.37 ± 0.78	−8.64 ± 0.24	18.10 ± 0.66	12.91 ± 0.38 cd	63.09 ± 1.12 e	35.27 ± 1.51 b
60:40 × CT1	41.43 ± 0.66	−9.25 ± 0.30	19.11 ± 1.51	16.50 ± 1.58 ab	75.81 ± 4.78 de	54.15 ± 6.06 a
40:60 × CT1	40.44 ± 0.43	−9.17 ± 0.79	18.73 ± 2.18	17.47 ± 0.46 a	107.96 ± 4.36 b	64.14 ± 4.87 a
20:80 × CT1	42.74 ± 0.67	−9.84 ± 0.53	22.31 ± 1.33	13.96 ± 1.27 bc	147.49 ± 12.5 a	62.33 ± 9.31 a
80:20 × CT2	41.24 ± 0.87	−16.64 ± 0.82	21.21 ± 1.63	10.80 ± 0.60 de	61.91 ± 4.46 e	29.58 ± 2.38 b
60:40 × CT2	42.64 ± 0.58	−18.17 ± 0.43	24.30 ± 0.84	10.59 ± 0.89 de	65.70 ± 2.94 e	26.54 ± 1.70 b
40:60 × CT2	42.95 ± 0.82	−17.18 ± 0.58	22.75 ± 1.09	9.01 ± 0.17 e	91.04 ± 0.28 cd	30.71 ± 4.81 b
20:80 × CT2	46.33 ± 0.90	−18.91 ± 0.20	26.69 ± 0.85	11.62 ± 1.09 cde	104.42 ± 0.55 bc	60.60 ± 9.13 a
	ns	ns	ns	*	*	*

Asterisks indicate a statistically significant difference (***: *p* < 0.001; **: *p* < 0.01; *: *p* < 0.05; ns: non-significant, *p* ≥ 0.05). For each source of variance, different letters within each column indicate statistically different groups according to the Duncan’s post-hoc test (*p* = 0.05). The factor “Cut” was analyzed using an independent Student’s t-test. Data are mean values ± standard error, *n* = 3.

**Table 5:** Analysis of variance and mean comparisons for the relative abundance of major aroma volatiles in greenhouse basil cultivated under four NO_3_^−^:Cl^−^ ratios (R) in the nutrient solution and harvested in two successive cuts (CT).

Source of Variance	β-myrcene	Eucalyptol	2-hexenal	β-ocimene	1-octen-3-ol	β-linalool	Trans-α-bergamotene	Eugenol
	(%)	(%)	(%)	(%)	(%)	(%)	(%)	(%)
NO_3_^−^:Cl^−^ ratio (R)								
80:20	1.55 ± 0.05 b	27.83 ± 0.67 a	2.09 ± 0.42	5.18 ± 0.67	5.53 ± 0.45	26.52 ± 1.23	9.43 ± 0.98 bc	2.79 ± 0.20 c
60:40	2.07 ± 0.29 a	24.36 ± 1.46 b	2.28 ± 0.57	5.05 ± 0.26	6.05 ± 0.39	28.45 ± 1.00	11.62 ± 1.15 ab	4.23 ± 0.16 a
40:60	2.31 ± 0.14 a	28.64 ± 1.49 a	2.39 ± 0.60	5.35 ± 0.43	6.24 ± 0.52	28.17 ± 0.89	7.82 ± 1.09 c	3.51 ± 0.45 b
20:80	2.60 ± 0.26 a	24.82 ± 0.77 b	3.69 ± 0.39	5.49 ± 0.15	4.70 ± 0.47	26.19 ± 0.57	12.37 ± 0.59 a	3.52 ± 0.16 b
	**	*	ns	ns	ns	ns	**	***
Cuts (CT)								
CT1	1.99 ± 0.21	25.16 ± 1.11	1.94 ± 0.38	5.81 ± 0.28	5.58 ± 0.30	27.67 ± 0.79	11.12 ± 0.99	3.71 ± 0.26
CT2	2.27 ± 0.15	27.66 ± 0.58	3.28 ± 0.28	4.73 ± 0.20	5.68 ± 0.41	26.99 ± 0.60	9.50 ± 0.60	3.32 ± 0.20
t-value	ns	ns	**	**	ns	ns	ns	ns
R × CT								
80:20 × CT1	1.48 ± 0.05 d	27.33 ± 0.76	1.37 ± 0.54	6.38 ± 0.90	4.80 ± 0.32	26.67 ± 2.30	10.25 ± 1.47	2.41 ± 0.16 d
60:40 × CT1	1.51 ± 0.04 d	21.36 ± 0.94	2.14 ± 1.25	5.27 ± 0.08	5.71 ± 0.68	27.21 ± 1.81	13.60 ± 1.27	4.14 ± 0.21 a
40:60 × CT1	2.06 ± 0.03 bcd	28.40 ± 2.91	1.30 ± 0.44	6.02 ± 0.67	6.14 ± 0.91	29.95 ± 0.71	7.32 ± 2.06	4.44 ± 0.40 a
20:80 × CT1	2.93 ± 0.49 a	23.57 ± 0.97	2.94 ± 0.46	5.56 ± 0.32	5.67 ± 0.24	26.86 ± 1.07	13.30 ± 0.79	3.83 ± 0.15 ab
80:20 × CT2	1.62 ± 0.08 cd	28.33 ± 1.20	2.81 ± 0.23	3.99 ± 0.17	6.26 ± 0.63	26.37 ± 1.52	8.60 ± 1.41	3.16 ± 0.19 bc
60:40 × CT2	2.63 ± 0.34 ab	27.36 ± 0.86	2.41 ± 0.25	4.84 ± 0.54	6.39 ± 0.44	29.68 ± 0.42	9.65 ± 1.08	4.31 ± 0.26 a
40:60 × CT2	2.56 ± 0.19 ab	28.89 ± 1.60	3.47 ± 0.65	4.68 ± 0.14	6.34 ± 0.71	26.39 ± 0.52	8.31 ± 1.21	2.59 ± 0.10 cd
20:80 × CT2	2.28 ± 0.01 abc	26.07 ± 0.70	4.43 ± 0.01	5.42 ± 0.11	3.73 ± 0.36	25.51 ± 0.15	11.44 ± 0.49	3.22 ± 0.06 bc
	*	ns	ns	ns	ns	ns	ns	***

Asterisks indicate a statistically significant difference (***: *p* < 0.001; **: *p* < 0.01; *: *p* < 0.05; ns: non-significant, *p* ≥ 0.05). For each source of variance, different letters within each column indicate statistically different groups according to the Duncan’s post-hoc test (*p* = 0.05). The factor “Cut” was analyzed using an independent Student’s t-test. Data are mean values ± standard error, *n* = 3.

**Table 6:** Analysis of variance and mean comparisons for the stomata and glands traits in leaves of basil cultivated under different NO_3_^−^:Cl^−^ ratios in the nutrient solution.

Source of Variance	Abaxial Epidermis Stomata Frequency	Abaxial Epidermis: Stomata Area	Adaxial Epidermis Stomata Frequency	Adaxial Epidermis Stomata Area	Adaxial Epidermis Gland Frequency
	(n/mm^2^)	(mm^2^)	(n/mm^2^)	(mm^2^)	(n/mm^2^)
NO_3_^−^:Cl^−^ ratio					
80:20	93.52 ± 6.34	470.5 ± 11.4 a	151.13 ± 8.40 a	363.1 ± 9.84 a	9.125 ± 0.88 a
60:40	93.70 ± 5.00	443.2 ± 14.6 a	115.14 ± 9.01 b	377.0 ± 10.2 a	6.493 ± 0.65 b
40:60	105.63 ± 3.81	434.5 ± 13.7 a	107.19 ± 10.9 bc	330.1 ± 10.7 b	5.440 ± 0.84 b
20:80	100.19 ± 3.43	395.7 ± 11.0 b	85.99 ± 5.81 c	353.4 ± 8.83 ab	5.089 ± 0.52 b
	ns	***	***	**	*

Asterisks indicate a statistically significant difference (***: *p* < 0.001; **: *p* < 0.01; *: *p* < 0.05; ns: non-significant, *p* ≥ 0.05). For each source of variance, different letters within each column indicate statistically different groups according to the Duncan’s post-hoc test (*p* = 0.05). The factor “Cut” was analyzed using an independent Student’s t-test. Data are mean values ± standard error, *n* = 3.

**Table 7:** Analysis of variance and mean comparisons for the mesophyll traits in leaves of basil cultivated under different NO_3_^−^:Cl^−^ ratios in the nutrient solution.

Source of Variance	Lamina Thickness	Palisade Tissue	Spongy Tissue	Intercellular	Palisade Cell	Spongy Cell
	(mm)	Thickness (mm)	Thickness (mm)	Spaces (%)	Area (mm^2^)	Area (mm^2^)
NO_3_^−^:Cl^−^ ratio						
80:20	199.3 ± 5.73 c	64.58 ± 1.93 b	96.93 ± 4.45 b	32.62 ± 1.39 a	802.3 ± 26.1 c	146.3 ± 3.72 b
60:40	228.0 ± 7.63 b	70.42 ± 2.78 b	120.8 ± 4.81 a	33.30 ± 1.61 a	960.0 ± 46.1 b	155.1 ± 4.09 ab
40:60	255.5 ± 7.55 a	82.33 ± 2.77 a	130.3 ± 6.42 a	29.60 ± 1.56 ab	1262 ± 48.1 a	160.8 ± 4.36 a
20:80	213.3 ± 4.63 bc	69.37 ± 1.40 b	102.0 ± 3.49 b	26.81 ± 1.53 b	996.3 ± 37.3 b	163.5 ± 4.02 a
	***	***	***	**	***	*

Asterisks indicate a statistically significant difference (***: *p* < 0.001; **: *p* < 0.01; *: *p* < 0.05; ns: non-significant, *p* ≥ 0.05). For each source of variance, different letters within each column indicate statistically different groups according to the Duncan’s post-hoc test (*p* = 0.05). The factor “Cut” was analyzed using an independent Student’s t-test. Data are mean values ± standard error, *n* = 3.

## Supplementary Materials

The following are available online at https://www.mdpi.com/2079-7737/9/7/158/s1, Figure S1. Scree plot of the eigenvalues of the principal components; Figure S2. Correlogram (Pearson) of the quantitative variables measured in the two successive harvests, CT1 and CT2. Pairwise correlations are color-mapped according to the color scale of the bar on the right-hand side. Asterisks indicate statistically significant correlations (*: *p* < 0.05; **: *p* < 0.01).
